# SNX3 Promotes Doxorubicin-Induced Cardiomyopathy by Regulating GPX4-Mediated Ferroptosis

**DOI:** 10.7150/ijms.95466

**Published:** 2024-06-17

**Authors:** Shuai Huang, Fan Zou, Hao Zhou, Jinyuan He

**Affiliations:** 1 Department of Cardio-Thoracic Surgery, The Third Affiliated Hospital, Sun Yat-Sen University, 510630, Guangzhou, China.; 2 School of Medicine, Southern University of Science and Technology, Shenzhen, Guangdong, China.

**Keywords:** SNX3, doxorubicin, GPX4, ferroptosis.

## Abstract

The complete molecular mechanism underlying doxorubicin-induced cardiomyopathy remains incompletely elucidated. In this investigation, we engineered mice with cardiomyocyte-specific *sorting nexin 3* knockout (*SNX3^Cko^*) to probe the potential protective effects of *SNX3* ablation on doxorubicin-triggered myocardial injury, focusing on GPX4-dependent ferroptosis. Our findings indicate that *SNX3* deletion normalized heart contractile/relaxation function and thwarted the escalation of cardiac injury biomarkers following doxorubicin exposure. Additionally, *SNX3* deletion in the heart mitigated the inflammatory response and oxidative stress in the presence of doxorubicin. At the molecular level, the detrimental effects of doxorubicin-induced cell death, endoplasmic reticulum (ER) stress, and mitochondrial dysfunction were alleviated by SNX3 deficiency. Molecular analysis revealed the activation of GPX4-mediated ferroptosis by doxorubicin, whereas loss of SNX3 prevented the initiation of GPX4-dependent ferroptosis. Furthermore, treatment with erastin, a ferroptosis inducer, markedly reduced cell viability, exacerbated ER stress, and induced mitochondrial dysfunction in *SNX3*-depleted cardiomyocytes upon doxorubicin exposure. In summary, our results demonstrate that SNX3 deficiency shielded the heart from doxorubicin-induced myocardial dysfunction by modulating GPX4-associated ferroptosis.

## Introduction

Doxorubicin-induced cardiotoxicity poses a significant challenge in cancer treatment, given its potential to inflict lasting harm on the heart muscle, culminating in heart failure and other cardiovascular complications 1. The utilization of this potent chemotherapy drug is frequently curtailed by its detrimental impact on cardiac health 2. The cardiac repercussions of Doxorubicin primarily stem from its capacity to engender reactive oxygen species (ROS) and provoke oxidative stress within cardiac cells 3, 4. This oxidative burden disrupts cellular operations, compromises mitochondrial function, and sparks inflammatory cascades 5-7, ultimately resulting in cardiomyocyte demise and dysfunction. Moreover, Doxorubicin can disrupt the expression of genes crucial for cardiac contractility and energy metabolism, exacerbating cardiac impairment 8, 9.

Various strategies have been posited to alleviate Doxorubicin-induced cardiotoxicity, including the deployment of cardioprotective agents like dexrazoxane 10, which can mitigate oxidative stress and shield the heart from harm during chemotherapy. Alternately, interventions encompass the use of antioxidants such as N-acetylcysteine 11 and coenzyme Q10 12 to counteract ROS effects and uphold cardiac function. Concurrently, research endeavors are underway to devise targeted therapies capable of specifically forestalling or reversing Doxorubicin's cardiotoxic repercussions while upholding its anticancer efficacy. In essence, a profound comprehension of the molecular mechanisms underpinning Doxorubicin-induced cardiotoxicity is imperative for crafting efficacious preventive and therapeutic measures to curtail the risk of cardiac injury among cancer patients undergoing this chemotherapy regimen.

Sorting Nexin 3 (SNX3) assumes a pivotal role in cardiac injury by regulating diverse cellular processes 13. Studies suggest that SNX3 governs endosomal trafficking, protein sorting, and membrane dynamics in cardiomyocytes 14-16. In the realm of cardiac injury, SNX3 influences pathways linked to inflammation, oxidative stress, apoptosis, and mitochondrial function 17-19. Through its modulation of these mechanisms, SNX3 can either exacerbate or alleviate cardiac damage, depending on the specific context. Unraveling the precise molecular mechanisms underlying SNX3's actions in cardiac injury is crucial for devising targeted therapies to address heart-related conditions effectively. Delving deeper into the complex interplay between SNX3 and cardiac pathophysiology shows potential for propelling cardiovascular research forward and enhancing therapeutic strategies.

GPX-dependent ferroptosis, involving the action of glutathione peroxidase 4 (GPX4), plays a critical role in cardiac injury, a condition characterized by iron-dependent lipid peroxidation 20, 21. This form of regulated cell death, known as ferroptosis, is associated with various pathological states, including cardiovascular diseases 22, 23. GPX4 serves as a crucial enzyme that shields cells from lipid peroxidation and the ensuing ferroptotic cell demise by reducing lipid hydroperoxides 24-26. Perturbations in GPX4 and the ferroptotic pathway within the context of cardiac injury can contribute to myocardial harm, inflammation, and oxidative stress, ultimately resulting in heart dysfunction and failure 27-30. A comprehensive understanding of the molecular mechanisms governing GPX iron death in cardiac injury is imperative for devising precise therapies aimed at safeguarding the heart from ferroptosis-induced damage.

Current investigations in the realm of GPX iron death and cardiac injury are primarily focused on delineating the specific pathways through which ferroptosis impacts myocardial damage and exploring potential therapeutic avenues to modulate this process. Studies are underway to elucidate GPX4's role in shielding cardiomyocytes from ferroptotic cell death and to assess the repercussions of disrupted iron metabolism on cardiac performance 31-33. Researchers are also exploring the utility of pharmacological agents targeting the ferroptotic pathway, including ferroptosis inhibitors and iron chelators, as potential interventions to prevent or alleviate cardiac injury 34, 35. Moreover, endeavors are ongoing to pinpoint novel biomarkers of ferroptosis in cardiac tissue and to develop non-invasive imaging techniques for monitoring ferroptosis progression in the heart 36-38.

In conclusion, the investigation of GPX iron death in cardiac injury represents a promising avenue of research with significant implications for the advancement of innovative therapies aimed at shielding the heart from ferroptosis and enhancing outcomes for individuals with cardiovascular ailments. Our study aims to delve into the role and molecular mechanisms of SNX3 in regulating doxorubicin-induced cardiomyopathy, with a specific focus on ferroptosis.

## Methods

### Ethical statement

The present study was conducted in accordance with the Declaration of Helsinki and the guidelines of the Ethics Committee of Sun Yat-Sen University, Guangzhou, China. All experimental protocol were approved by Ethics Committee of Sun Yat-Sen University, Guangzhou, China. The ethical approval number is SYS-2022-3J05.

### Animals

C57BL/6J mice were obtained from Shanghai Laboratory Animal Company (SLAC) and treated as indicated in the figure legends. To induce doxorubicin-mediated myocardial injury, cardiomyocyte-specific SNX3 knockout (*SNX3^Cko^*) mice were purchased from GemPharmatech and administered a single intraperitoneal injection of doxorubicin (15 mg/kg). After 28 days, cardiac tissues were rapidly harvested and either snap-frozen in liquid nitrogen or fixed in 4% paraformaldehyde for subsequent analyses.

### Echocardiography

Transthoracic echocardiography was conducted on conscious mice utilizing a Vevo 2100 system equipped with a 40MHz linear transducer from FUJIFILM VisualSonics in Toronto, Canada, following standard procedures 39. The M-mode echocardiogram was captured from the short-axis perspective of the left ventricle (LV) at the midpapillary muscle level with a sweep speed of 200 mm/sec. Key cardiac parameters including inter-ventricular septal thickness at end-diastole (IVSd), LV internal diameter at end-diastole (LVIDd), LV posterior wall thickness at end-diastole (LVPWd), and LV internal diameter at end-systole (LVIDs) were measured from this view. These parameters were then utilized to calculate percent fractional shortening (FS), relative wall thickness (RWT), and left ventricle mass (LV Mass) for the assessment of cardiac contractility and left ventricle morphology 40.

### Histological assessment

Heart tissue samples underwent standard processing procedures as described previously. Following fixation in 10% neutral formalin, the samples were embedded in paraffin. Subsequent to this, serial sections measuring 4 µm in thickness or mirror-image sections were prepared and stained using either hematoxylin and eosin. The evaluation of histopathological observations was conducted by a skilled pathologist, who utilized Knodell's and Scheuer's scoring systems for assessment.

### Cardiomyocyte isolation

Adult rat ventricular myocytes (ARVM) were obtained from male Sprague-Dawley rats in accordance with established procedures. Initially, rats weighing 300-500g and aged 4-6 months were anesthetized using isoflurane and then humanely euthanized through cervical dislocation. Following the removal of the heart from the thoracic cavity, it was promptly submerged in ice-cold Krebs-Henseleit (KH) Buffer 41. Subsequently, the lungs, thyroid, and pericardial fat were excised, the aorta was cannulated, and the heart was perfused with KH buffer at 37°C utilizing a Langendorff apparatus 42. The KH buffer's elevated calcium concentration was crucial in sustaining the heart's rhythmic contractions while simultaneously purging the blood. Once the effluent became clear, the heart underwent perfusion with a low-calcium buffer (LoCa^2+^) enriched with 12-15µM CaCl_2_, 120mM NaCl, 5.4mM KCl, 5mM MgSO_4_, 5mM pyruvate, 20mM glucose, 20mM taurine, 10mM HEPES, and 5mM nitrilotriacetic acid. This alteration in calcium levels, alongside the presence of nitrilotriacetic acid, induced cardiac arrest, shielding the cardiomyocytes from hypercontracture during the subsequent 5-minute perfusion period. The heart was subsequently subjected to perfusion with an oxygenated enzymatic solution comprising 1mg/ml Collagenase II and 0.6mg/ml Hyaluronidase (C+H) in an enzyme (Enz) buffer (12-15µM CaCl_2_, 120mM NaCl, 5.4mM KCl, 5mM MgSO_4_, 5mM pyruvate, 20mM glucose, 20mM taurine, 10mM HEPES, 150µM Ca^2+^) for enzymatic digestion and isolation of cardiomyocytes from the scaffold. The heart underwent perfusion in this enzymatic solution for 10 minutes before being transferred to a plate containing fresh C+H buffer for subsequent processing. The process began by extracting the left ventricles, which were finely chopped and placed in a clean 50mL tube along with fresh C+H buffer. The tube was then agitated at 35°C for 5 minutes. Subsequently, the mixture was filtered through gauze to gather the isolated cardiomyocytes supernatant. The residual tissue was then moved to a falcon tube with additional C+H buffer for a second digestion cycle, undergoing agitation for 30 minutes at 35°C. After filtering the mixture once more through gauze, the supernatant was collected. The supernatant, containing the cardiomyocytes, underwent centrifugation at 700 rpm for 1 minute to yield a cell pellet, with the supernatant being removed. The cell pellet was resuspended in fresh Enz buffer, preparing it for subsequent procedures. Cardiomyocytes isolated through this method maintained their characteristic rod-shaped morphology, exhibited calcium tolerance, and mostly remained inactive unless provoked, showcasing robust stability in an artificial setting.

### LDH leakage measurement

To quantify the extent of lactate dehydrogenase (LDH) release from cardiomyocytes (CMs) compromised by injury, we employed a commercial LDH Cytotoxicity Detection Kit (Takara Bio, MK401), following the protocol provided. Subsequent to the specified CM death induction, we subjected the culture media to low-speed centrifugation at 1,000 g for a brief 5-minute period at 4°C, which facilitated the retrieval of the supernatant necessary for assessing LDH release. This supernatant was then reacted with the kit's provided mixture at room temperature (RT) for half an hour. Absorbance readings at 490 nm were subsequently obtained using a multi-well spectrophotometer. Data interpretation was based on a comparative analysis between the experimental absorbance values and those of a control group, which were obtained from CMs treated with a 1% Triton X-100 solution for a full day to ensure maximum LDH release. The final data were expressed in terms of the multiple of change relative to the control group's readings.

### TUNEL staining and quantification in the heart

Myocardial apoptosis in the heart was assessed through Terminal deoxynucleotidyl transferase dUTP nick end labeling (TUNEL) staining. Frozen heart sections were air-dried and fixed in 4% paraformaldehyde (PFA) at room temperature for 1 hour. Following PBS washing, the sections were permeabilized with 1% Triton X-100 at room temperature for 1 hour. Subsequently, the heart sections underwent another round of PBS washing and were then treated with TUNEL staining buffer at 37°C for 1 hour, following the guidelines outlined in the commercial kit manual (Sigma-Aldrich, 12156792910).

To visualize myocytes and nuclei, membrane staining was performed using Alexa Fluor™ 488-conjugated wheat germ agglutinin (WGA) at a concentration of 20 μg/ml at room temperature for 1 hour, and nuclear staining was achieved with 4',6-diamidino-2-phenylindole (DAPI) at 1 μg/mL at room temperature for 5 minutes. The mounted heart sections were preserved using a gold anti-fade mounting solution. Apoptotic cardiomyocytes (CMs) were identified as TUNEL-positive nuclei exhibiting red fluorescence.

Each mouse contributed one heart section for analysis. Ten fields from each heart section were randomly captured using an Olympus upright Epi-Fluorescence Microscope BX51. Approximately 1 × 10^4 cells from each heart section were quantified utilizing ImageJ software. The findings were presented as the percentage of TUNEL-positive myocardial cells relative to the total myocardial cell count in each heart section. Representative images were selected based on quality and accuracy to best represent the group average across all available data.

### Reverse transcribed quantitative polymerase chain reaction (RT-qPCR)

Total RNA was isolated utilizing the TRIzol reagent (Invitrogen) following the manufacturer's guidelines. Subsequently, cDNA was synthesized through reverse transcription employing the FastQuant RT kit (Tiangen, Beijing, China). The resultant cDNA was then utilized with the SuperReal PreMix Plus (Tiangen) on the ABI ViiA 7 Dx machine (Applied Biosystems, Foster City, CA, United States) for analysis. Relative RNA expression levels were assessed using the comparative threshold cycle (2^-ΔΔCT^) method.

### Immunofluorescence (IF) staining

Isolated adult rat ventricular myocytes (ARVMs) were fixed with 4% paraformaldehyde (PFA) in phosphate-buffered saline (PBS) for 15 minutes at room temperature. Following fixation, the cells underwent three washes with 1X PBS before being treated with 0.1% Triton-X in PBS for 15 minutes to aid in cell penetration. After another three washes in 1X PBS, the samples were blocked in a solution of 1% bovine serum albumin (BSA) with 5% filtered goat serum in PBS, supplemented with 0.1% Triton-X, for 1 hour at room temperature. Subsequently, the cells were subjected to primary antibody incubation overnight at 4°C. After the overnight incubation, the samples were washed three times with 1X PBS before secondary antibodies (donkey anti-mouse AF488 and goat anti-rabbit AF546) or AF546 Phalloidin were added and incubated at room temperature for 1 hour. Following this, the samples underwent another three washes in 1X PBS and were counterstained with DAPI before mounting on glass slides using appropriate mounting media. Imaging of the samples was carried out using either a Zeiss LSM-780 confocal microscope or a Leica Stellaris 8 microscope. Deconvolution of images was performed using the Leica Stellaris 8 microscope's built-in Stellaris Lightening platform. To validate the specificity of the labeling, negative controls using only secondary antibodies were conducted on control adult rat cardiomyocytes following the same experimental procedures. Representative images were selected for illustration. To ensure objectivity, sample preparation, imaging, and image analysis were conducted in parallel by a minimum of two individuals.

### Statistical analysis

Statistical analysis was conducted using GraphPad Prism 8.0 software (GraphPad Prism Software, Inc., San Diego, CA). The distribution of the samples was assessed through either the Kolmogorov-Smirnov or Shapiro-Wilk normality test. When analyzing parametric data, differences between two groups were determined using a two-tailed unpaired Student's t-test. In cases where more than two groups were compared, one-way or two-way ANOVA followed by the Bonferroni post hoc test was employed. Nonparametric data analysis involved the use of the Mann-Whitney U test with the exact method to identify differences between two groups. Percentages were compared using the chi-square test. All p values were two-tailed, with values below 0.05 considered statistically significant.

## Results

### Deletion of *SNX3* mitigates Doxorubicin-induced cardiac dysfunction

**Figure 1:**
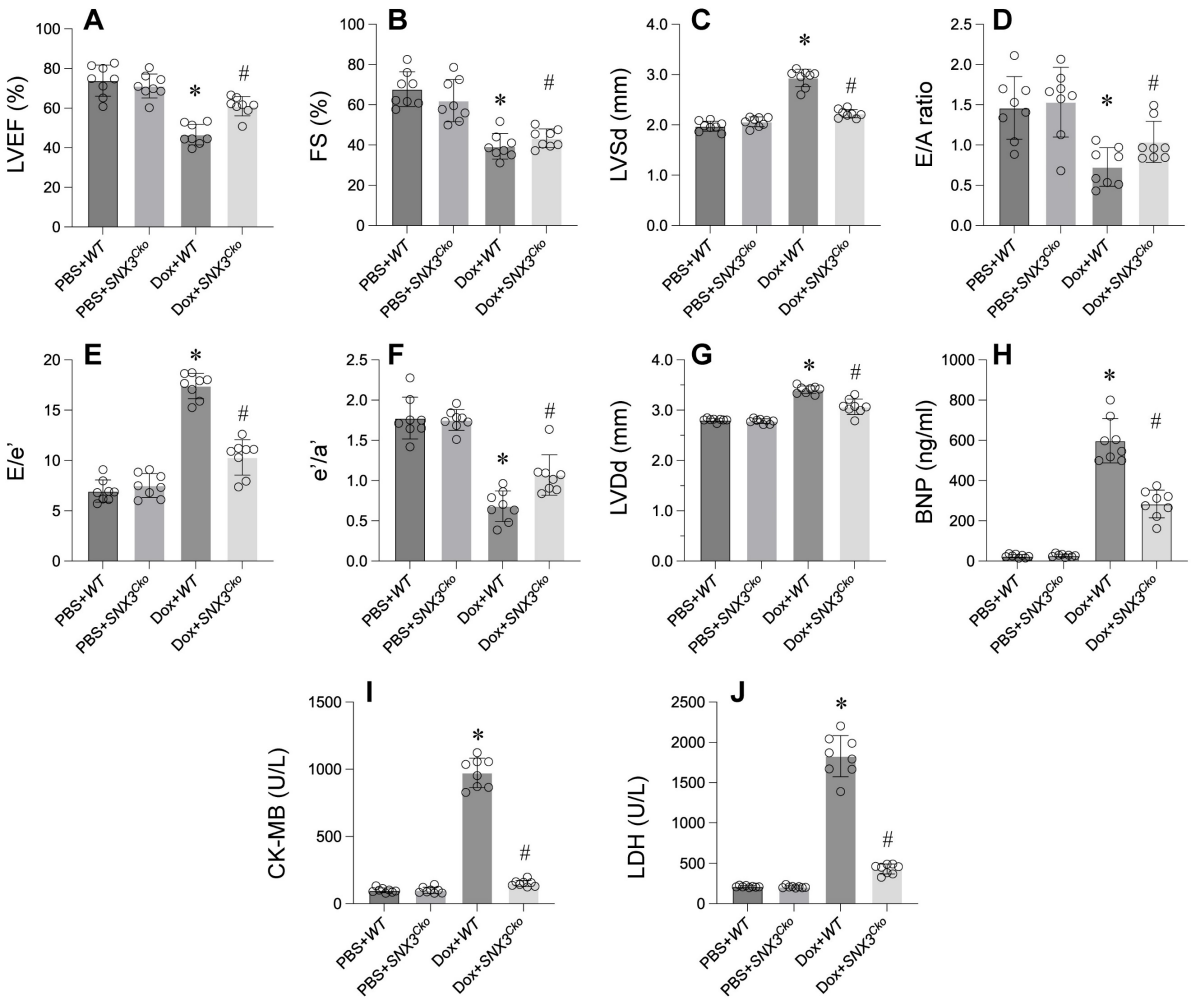
** Deletion of *SNX3* Mitigates Doxorubicin-Induced Cardiac Dysfunction. A-G.** Heart function was determined by echocardiography. **H-J.** ELISA kit was used to determine the concentration of BNP, CK-MB and LDH. *p<0.05 vs. PBS+WT group, #p<0.05 vs. Dox+WT group.

In exploring the impact of SNX3 on doxorubicin-triggered myocardial injury, we engineered cardiomyocyte-specific *SNX3* knockout (*SNX3^Cko^*) mice. Subsequent assessment of cardiac function via echocardiography unveiled a significant suppression of contractile parameters, including LVEF, FS, and LVSd, following doxorubicin administration compared to PBS-treated counterparts (Figure 1A-G). Moreover, doxorubicin perturbed diastolic functions, as evidenced by disruptions in E/A, E/e', e'/a', and LVDd relative to baseline measurements (Figure 1A-G). Notably, the absence of SNX3 notably restored the heart's contractile and relaxation parameters.

Furthermore, ELISA analysis revealed a substantial increase in BNP, CK-MB, and LDH concentrations in response to doxorubicin treatment (Figure 1H-J). Conversely, doxorubicin-exposed *SNX3^Cko^* mice exhibited a reduction in these cardiac biomarkers, bringing their levels closer to normalcy (Figure 1H-J). In summary, these findings underscore the protective role of *SNX3* deletion against doxorubicin-induced myocardial damage.

### Deletion of *SNX3* alleviates myocardial inflammation and oxidative stress

**Figure 2:**
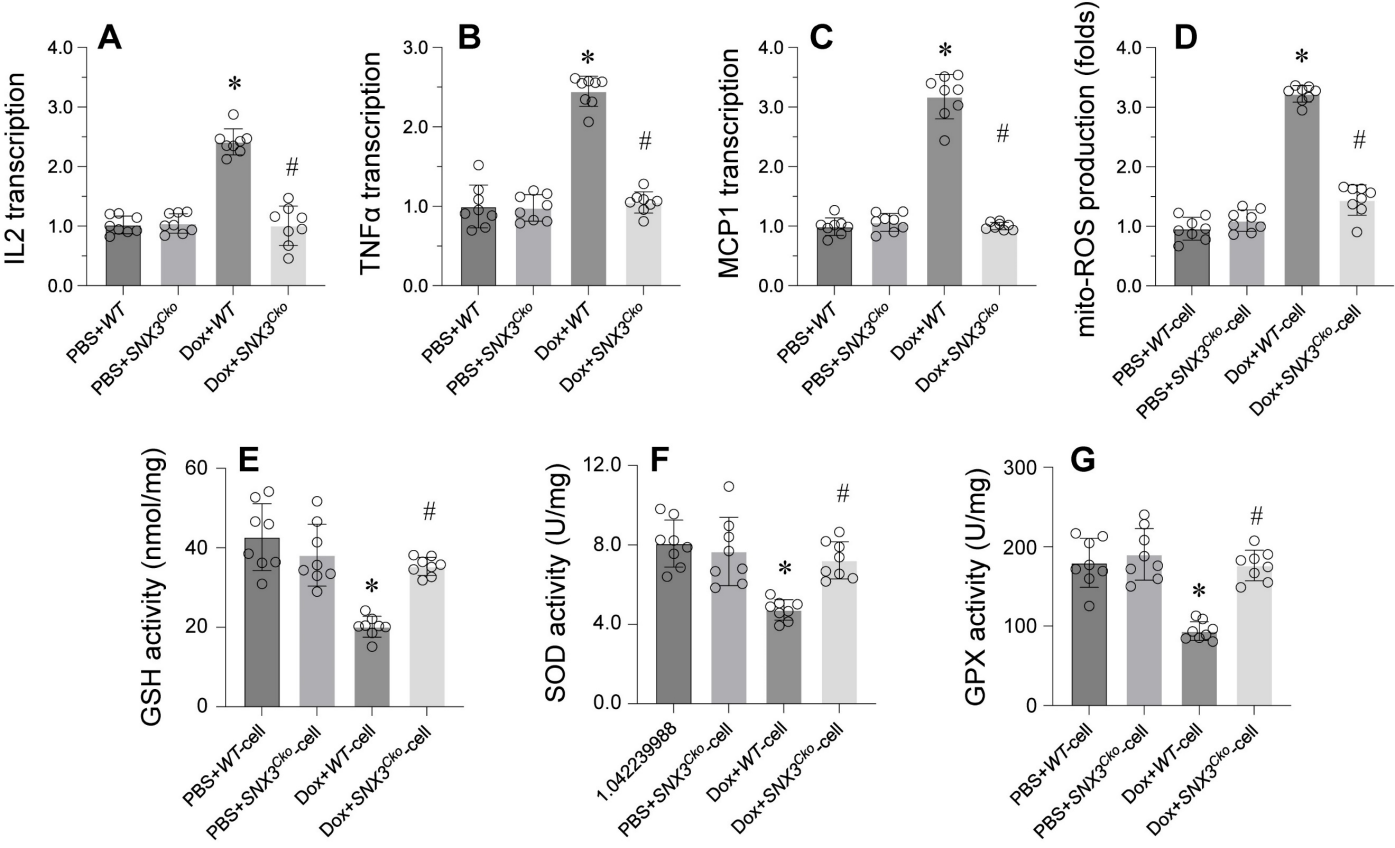
** Deletion of *SNX3* Alleviates Myocardial Inflammation and Oxidative Stress. A-C.** Western blots were used to observe the protein expression of IL-2, MCP1 and TNFα.** D.** Mitochondrial ROS production was detected by immunofluorescence.** E-G.** The concentrations of GSH, SOD and GPX were determined by ELISA. *p<0.05 vs. PBS+WT group or PBS+WT-cell group, #p<0.05 vs. Dox+WT-cell group.

Inflammation response and oxidative stress have emerged as key mechanisms underlying doxorubicin-induced myocardial damage. We sought to investigate whether *SNX3* deletion could mitigate myocardial inflammation and oxidative stress. Initially, we observed a rapid increase in the protein expression of IL-2, TNFα, and MCP1 in doxorubicin-treated mice (Figure 2A-C). Concurrently, there was an upregulation in mitochondrial ROS production following doxorubicin treatment (Figure 2D). Conversely, the levels of GSH, SOD, and GPX were notably reduced in response to doxorubicin exposure (Figure 2E-G).

Remarkably, *SNX3* deletion led to a significant decrease in the expression of IL-2, TNFα, and MCP1, indicating the pro-inflammatory role of SNX3 in doxorubicin-treated hearts (Figure 2A-C). Additionally, we found that *SNX3* ablation effectively reduced ROS generation (Figure 2D). Furthermore, the antioxidative enzymes GSH, SOD, and GPX were restored to near-normal levels upon *SNX3* deletion in the heart (Figure 2E-G).

In conclusion, these findings suggest that SNX3 deficiency alleviates the inflammation response and oxidative stress associated with myocardial damage.

### *SNX3* deletion attenuates cell apoptosis, ER stress, and mitochondrial dysfunction in the context of Doxorubicin-induced myocardial damage

At the molecular level, cell apoptosis, ER stress, and mitochondrial dysfunction have been identified as potential mechanisms contributing to doxorubicin-induced myocardial damage. To elucidate the role of SNX3 in cell apoptosis, ER stress, and mitochondrial dysfunction, we isolated cardiomyocytes from both wild-type (WT) mice and* SNX3^Cko^* mice and subjected them to doxorubicin treatment *in vitro*.

**Figure 3:**
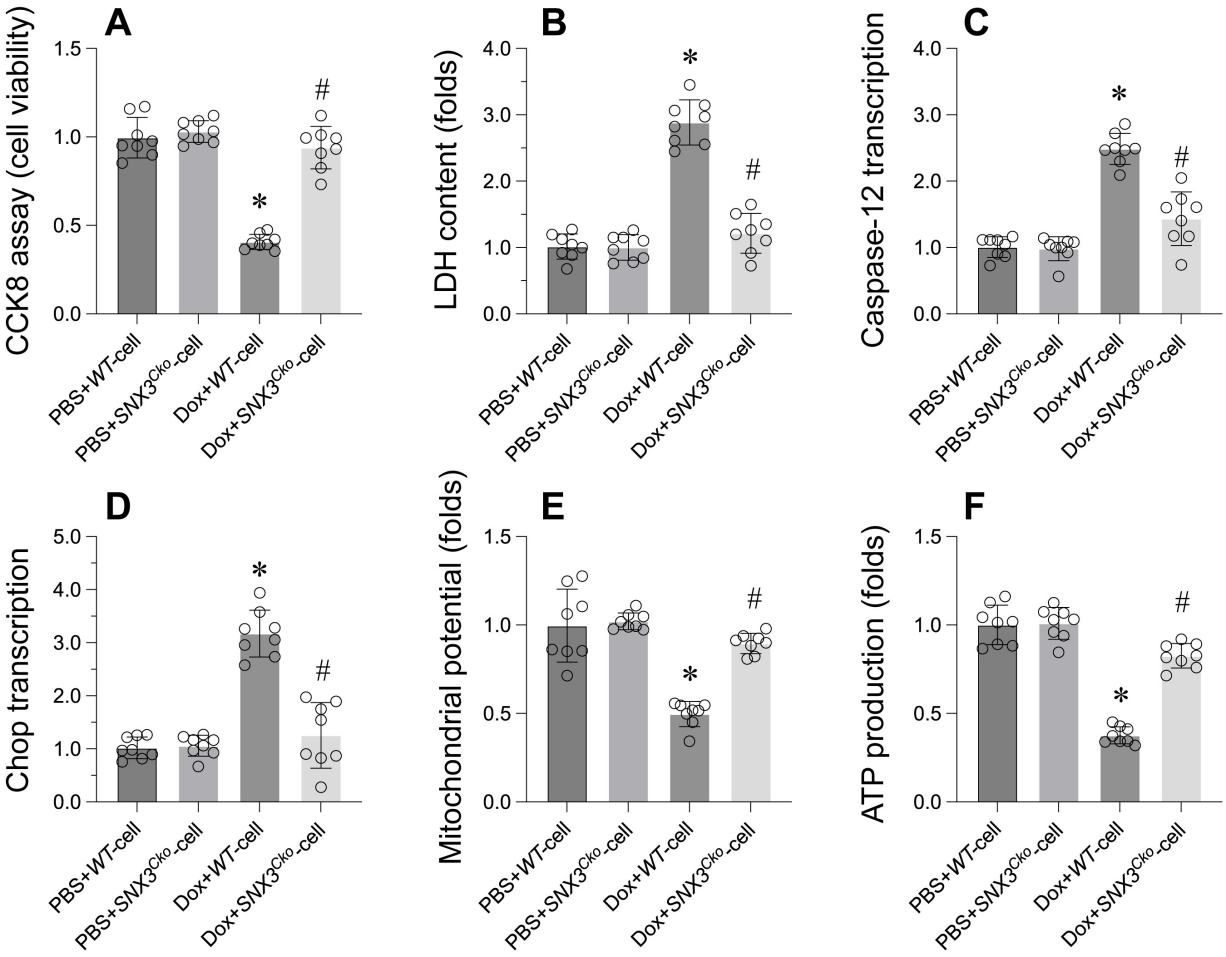
***SNX3* deletion Attenuates Cell Apoptosis, ER Stress, and Mitochondrial Dysfunction in the Context of Doxorubicin-Induced Myocardial Damage. A.** Cell viability was determined by CCK-8 assay. **B.** ELISA kit was used to measure the LDH content in the medium to evaluate the cell viability. **C-D.** qPCR assay was used to determine the transcription of caspase-12 and Chop in cardiomyocytes. **E.** Mitochondrial membrane potential was observed through JC-1 probe. **F.** ATP production was determined by ELISA. *p<0.05 vs. PBS+WT-cell group, #p<0.05 vs. Dox+WT-cell group.

The CCK-8 assay revealed a decrease in cardiomyocyte viability upon exposure to doxorubicin (Figure 3A). Furthermore, the LDH release assay confirmed that doxorubicin led to the release of LDH into the medium (Figure 3B). Intriguingly, cardiomyocytes isolated from *SNX3^Cko^* mice exhibited normalized cell viability and inhibited LDH release in the presence of doxorubicin (Figure 3A-B).

In terms of ER stress, we observed a rapid elevation in the transcription of caspase-12 and Chop in response to doxorubicin treatment (Figure 3C-D). Remarkably, *SNX3* deletion prevented the activation of ER stress-related markers (Figure 3C-D). Additionally, mitochondrial function was assessed by analyzing mitochondrial membrane potential and mitochondria-derived ATP production. Doxorubicin treatment was found to decrease mitochondrial membrane potential and suppress mitochondria-derived ATP production (Figure 3E-F). However, deletion of SNX3 maintained mitochondrial potential and promoted mitochondria-derived ATP generation (Figure 3E-F).

In summary, our findings demonstrate that *SNX3* deletion preserves cell viability, mitigates ER stress, and alleviates mitochondrial dysfunction in doxorubicin-treated cardiomyocytes.

### Suppression of GPX4-dependent ferroptosis by *SNX3* deletion in Doxorubicin-induced heart dysfunction

**Figure 4:**
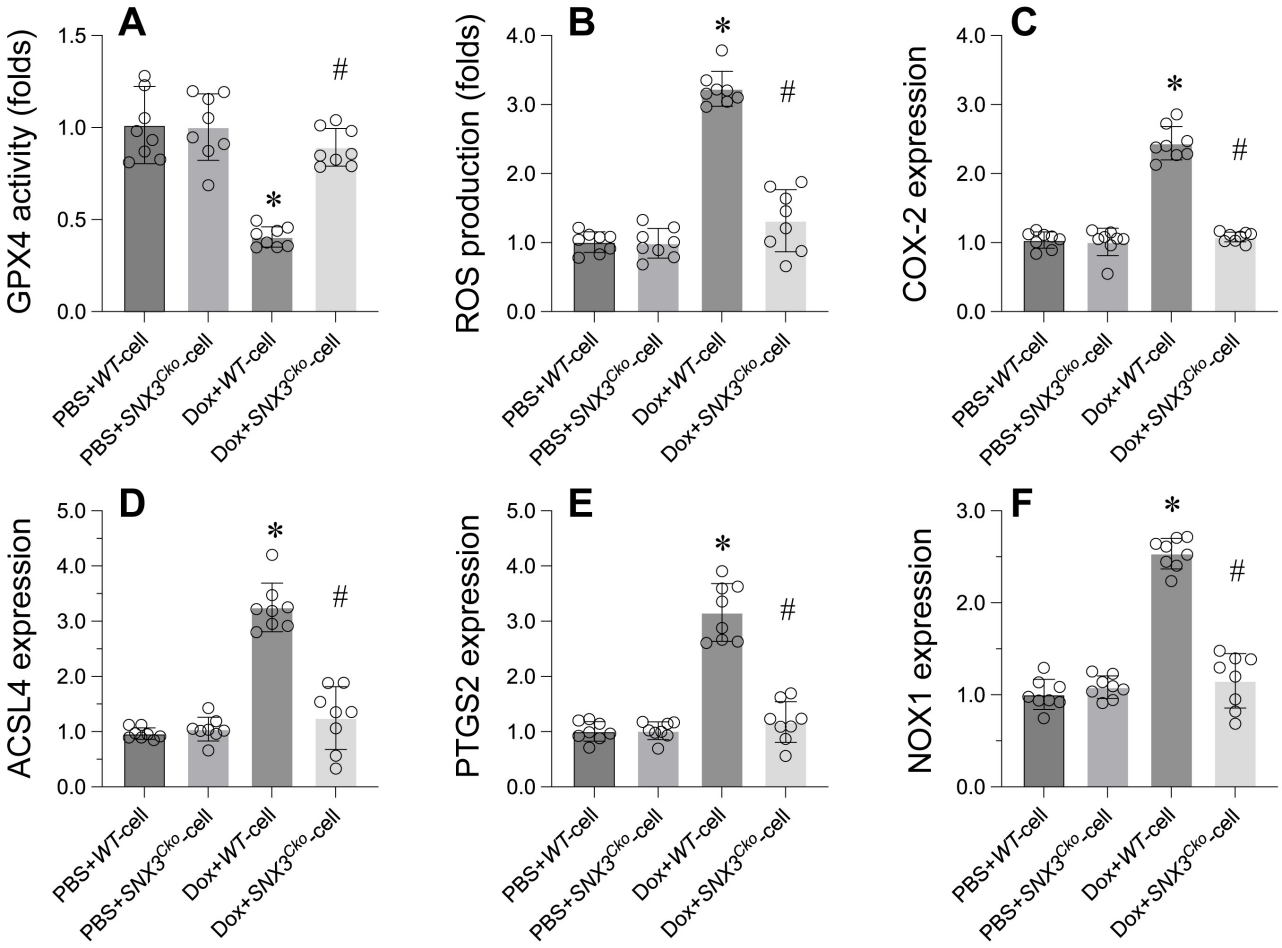
** Suppression of GPX4-Dependent Ferroptosis by *SNX3* Deletion in Doxorubicin-Induced Heart Dysfunction. A.** qPCR was used to analyze the transcription of GPX4 in cardiomyocytes.** B.** ROS production was detected by immunofluorescence. **C-E.** Western blots were used to determine the protein expression of COX-2, PTGS2, NOX1, and ACSL4. p<0.05 vs. PBS+WT-cell group, #p<0.05 vs. Dox+WT-cell group.

Ferroptosis has been identified as the primary cell death mechanism underlying doxorubicin-induced heart dysfunction. We investigated whether *SNX3* deletion could prevent ferroptosis in cardiomyocytes. Initially, GPX4, a key marker of ferroptosis, was significantly downregulated in doxorubicin-treated cardiomyocytes (Figure 4A). However, deletion of SNX3 normalized GPX4 transcription levels (Figure 4A). Additionally, ROS production was markedly increased by doxorubicin, but *SNX3* deletion prevented ROS accumulation in cardiomyocytes (Figure 4B). Furthermore, other ferroptosis-related markers such as COX-2, ACSL4, PTGS2, and NOX1 were upregulated by doxorubicin (Figure 4C-F). Interestingly, *SNX3* deletion normalized these parameters in the presence of doxorubicin (Figure 4C-F).

In conclusion, our data emphasize that SNX3 plays a crucial role in regulating GPX4-dependent ferroptosis in the context of doxorubicin-induced heart dysfunction.

### Revoking the protective effects of *SNX3* deletion on cardiomyocyte viability through ferroptosis reactivation

**Figure 5:**
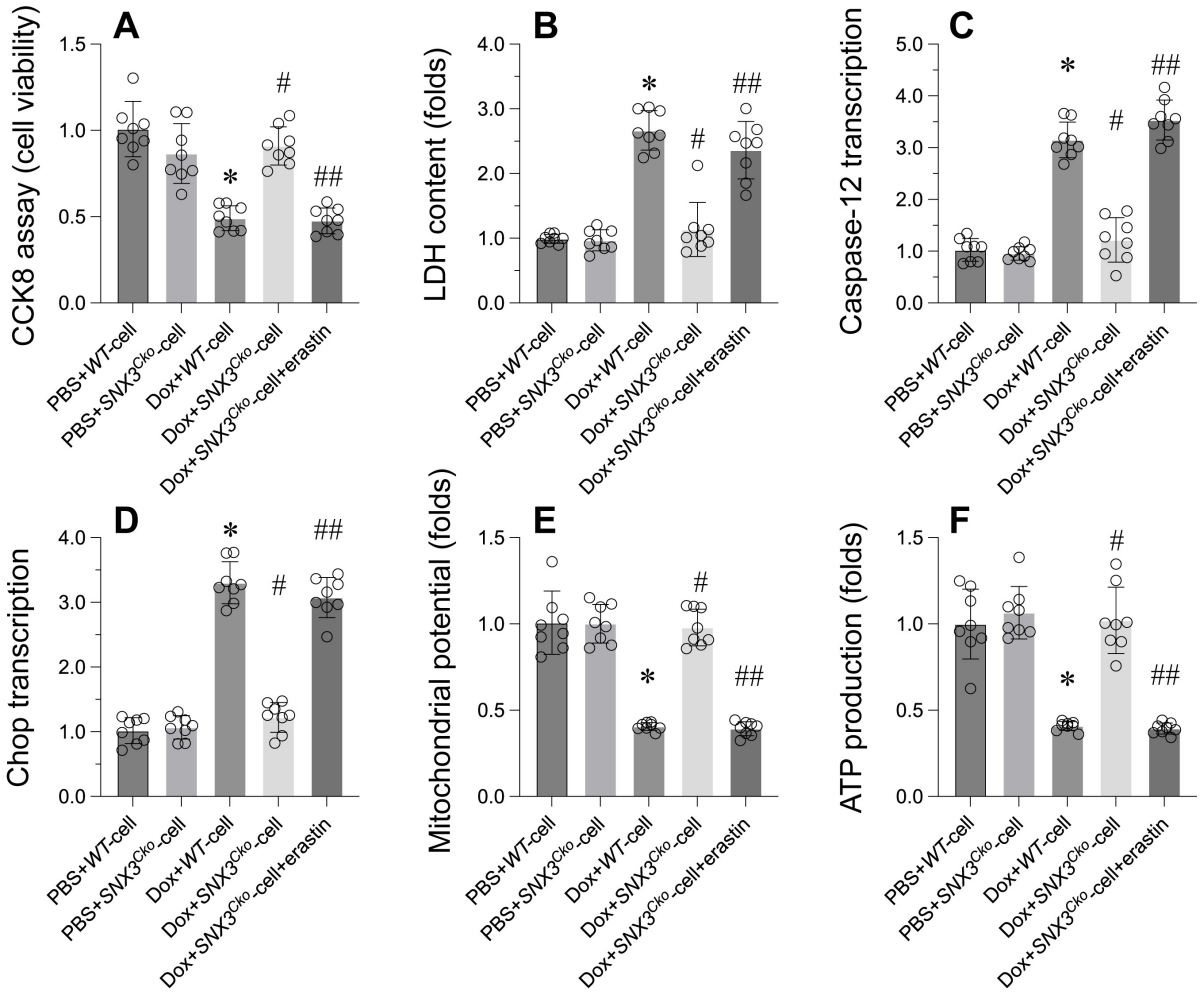
** Revoking the Protective Effects of SNX3 Deletion on Cardiomyocyte Viability through Ferroptosis Reactivation. A.** Cell viability was determined by CCK-8 assay. **B.** ELISA kit was used to measure the LDH content in the medium to evaluate the cell viability. **C-D.** qPCR assay was used to determine the transcription of caspase-12 and Chop in cardiomyocytes. **E.** Mitochondrial membrane potential was observed through JC-1 probe. **F.** ATP production was determined by ELISA. *p<0.05 vs. PBS+WT-cell group, #p<0.05 vs. Dox+WT-cell group, ##p<0.05 vs. Dox+*SNX3^Cko^*-cell group.

To investigate whether SNX3 modulates cardiomyocytes through ferroptosis, an inducer of ferroptosis was introduced to *SNX3*-deleted cardiomyocytes. Subsequently, cell viability, ER stress, and mitochondrial function were reassessed. As depicted in Figure 5A-B, erastin, a ferroptosis inducer, significantly diminished cell viability and increased LDH release compared to *SNX3*-deleted cardiomyocytes. Furthermore, ER stress-related parameters, including caspase-12 and Chop transcription levels, which had been normalized by *SNX3* deletion (Figure 5C-D), were once again upregulated following erastin treatment. Similarly, the disruption of mitochondrial membrane potential by erastin contrasted with the stabilization achieved by *SNX3* deletion in the presence of doxorubicin (Figure 5E). While *SNX3* deletion had facilitated ATP production during doxorubicin exposure, this effect was attenuated by erastin (Figure 5F).

In summary, our findings indicate that the protective mechanism mediated by *SNX3* deletion is intricately linked to the inhibition of ferroptosis in the context of doxorubicin-induced myocardial damage.

## Discussion

Doxorubicin, a potent chemotherapeutic agent utilized in the treatment of various cancers, faces significant limitations in clinical use due to its cardiotoxic effects, often resulting in cardiomyopathy and heart failure 43. Recent research has highlighted ferroptosis, a form of programmed cell death driven by iron-dependent lipid peroxidation, as a key player in doxorubicin-induced cardiac injury.

Ferroptosis is initiated by an imbalance in cellular redox status, characterized by the accumulation of reactive oxygen species (ROS) and depletion of glutathione (GSH), ultimately leading to lethal lipid peroxidation 44. Doxorubicin exacerbates this process by promoting iron accumulation and ROS generation in cardiomyocytes 45. It disrupts the mitochondrial electron transport chain, increases the labile iron pool, and compromises the heart's endogenous antioxidant defenses, including the function of glutathione peroxidase 4 (GPX4).

Numerous studies have highlighted the significant reduction in doxorubicin-induced cardiotoxicity through the inhibition of ferroptosis 46. Various approaches have been explored to achieve this, including iron chelation, lipid peroxidation inhibitors, and the upregulation of GPX4 47, 48. Furthermore, the use of ferroptosis inhibitors, such as liproxstatin-1, has demonstrated protective effects against doxorubicin-induced cardiac damage in preclinical models.

In addition to these findings, researchers have started to unravel the molecular pathways underlying doxorubicin-induced ferroptosis 30. Key signaling pathways, such as the p53 axis, have emerged as critical players in this process. Modulating p53 has been shown to impact the sensitivity to ferroptosis in the context of doxorubicin-induced cardiotoxicity.

The growing recognition of ferroptosis in doxorubicin-related cardiac injury opens up new possibilities for therapeutic interventions. Targeting specific ferroptosis mechanisms may offer a means to alleviate the cardiotoxic effects of doxorubicin, ultimately enhancing the well-being and survival rates of cancer patients undergoing chemotherapy. As research progresses, the development of therapies targeting ferroptosis holds promise for safer and more effective chemotherapeutic protocols.

Recent research has brought to light the diverse functions of Sorting Nexin 3 (SNX3) in the realms of chemotherapy and inflammation 49. SNX3 is recognized as a pivotal mediator of cellular responses to chemotherapeutic agents, impacting both drug sensitivity and resistance mechanisms 50. Furthermore, its role in modulating inflammatory pathways has been elucidated, underscoring its potential as a therapeutic target for inflammatory conditions and cancer therapy. There is a need for further investigation to delve into the intricate mechanisms governing SNX3's activities and its therapeutic relevance in these contexts.

Functionally, SNX3 belongs to the sorting nexin protein family, primarily involved in endosomal sorting and trafficking processes. Recent studies have unveiled the manifold roles of SNX3 in cellular damage, underscoring its importance across various pathophysiological conditions 51-53. SNX3 is instrumental in governing endosomal dynamics, membrane trafficking, and protein sorting within cells. In the context of cellular injury, SNX3 is implicated in crucial processes such as autophagy, apoptosis, and inflammation, interacting with a range of signaling molecules and cellular organelles to regulate cell survival and death pathways. Research indicates that SNX3 can impact cellular injury progression through diverse mechanisms, including its role in regulating the recycling of membrane receptors crucial for cell survival and apoptosis signaling.

Additionally, the role of SNX3 in endosomal trafficking has been associated with the modulation of inflammatory responses and the removal of damaged organelles, such as mitochondria, under conditions of cellular stress 14. Recent indications also point towards SNX3's potential involvement in regulating lipid metabolism and maintaining redox balance, influencing cellular reactions to oxidative stress and metabolic disturbances 18. Disruptions in SNX3 function have been linked to a range of diseases, including neurodegenerative disorders 54, cardiovascular conditions 13, and cancer 50, highlighting its significance in upholding cellular equilibrium.

The intricate molecular mechanisms through which SNX3 operates in cellular injury encompass its impact on endosomal trafficking, autophagy, apoptosis, inflammation, and metabolic control 55. A comprehensive understanding of SNX3's precise roles in diverse cellular environments and disease contexts is essential for uncovering its therapeutic possibilities. Further investigations into the interplay between SNX3 and pathways involved in cellular damage may reveal novel targets for therapeutic strategies across various pathological conditions. In the realm of addressing doxorubicin-induced cardiac injury, we are investigating a promising strategy by SNX3 and ferroptosis as critical therapeutic pathways. Through the strategic manipulation of these targets, novel approaches are being explored to reduce the cardiotoxic impact of doxorubicin and improve overall cardiac well-being.

SNX3's pivotal role in endosomal dynamics and cellular signaling offers a distinct opportunity for intervention in combating cardiac injury. Strategies focused on modulating SNX3 expression or activity have the potential to influence critical cellular processes implicated in cardiac damage, including autophagy, apoptosis, and inflammatory responses. Through the targeting of SNX3, researchers strive to optimize endosomal trafficking pathways, bolster cellular clearance mechanisms, and bolster cell survival under doxorubicin-induced stress conditions.

Considering the growing importance of ferroptosis in doxorubicin-induced cardiotoxicity, the inhibition of this iron-dependent form of cell death emerges as a promising therapeutic avenue 56, 57. By directing efforts towards key regulators of ferroptosis, such as lipid peroxidation and iron metabolism, scientists aim to prevent the harmful accumulation of reactive oxygen species and lipid peroxides within cardiomyocytes 58. This strategic approach seeks to uphold cellular membrane integrity, avert mitochondrial impairment, and ultimately shield the heart from the detrimental effects of doxorubicin-induced injury.

An integrated approach that concurrently targets SNX3 and ferroptosis presents an opportunity for synergistic therapeutic benefits. By capitalizing on the interplay between SNX3-mediated endosomal trafficking and ferroptosis pathways, we are striving to devise novel interventions that not only alleviate doxorubicin-induced cardiac injury but also enhance overall cardiac functionality and resilience. The development of specific inhibitors or activators that target SNX3 and ferroptosis pathways is crucial for translating these innovative intervention strategies into clinical practice.

Potential therapeutic agents for addressing doxorubicin-induced cardiac injury may include small molecules, peptides, or gene therapy approaches tailored to modulate SNX3 expression or regulate ferroptosis pathways. Thorough preclinical investigations utilizing cell culture models and animal studies are essential for assessing the efficacy and safety of SNX3 and ferroptosis-targeted interventions. Subsequent clinical trials can further validate the therapeutic promise of these strategies in human subjects, paving the way for the advancement of precision medicine tactics in managing doxorubicin-induced cardiotoxicity.

By harnessing the collective potential of targeting SNX3 and ferroptosis, we aim to transform the treatment landscape for doxorubicin-induced cardiac injury, offering renewed optimism for enhancing patient outcomes and quality of life.
